# Burnout, stress, and resilience in pediatric onco-hematology rehabilitation: results from the multicenter Rehabilitation Burnout Study (REBURN)

**DOI:** 10.1007/s00520-026-10692-0

**Published:** 2026-05-08

**Authors:** Giulia Zucchetti, Francesca Rossi, Elisa Marconi, Monica Valle, Chiara Battaglini, Federica Nota, Riccardo Casalaz, Paolo Colavero, Maria Montanaro, Federica Maio, Cinzia Favara Scacco, Mario Cardano, Alessandro Gennaro, Dorella Scarponi, Paola Quarello, Johanna M. C. Blom, Franca Fagioli

**Affiliations:** 1 Paediatric Onco-Hematology, Stem Cell Transplantation and Cellular Therapy Division, Regina Margherita Children’s Hospital, Turin, Italy; 2 Rehabilitation Service, Public Health and Pediatric Sciences Department, Regina Margherita Children’s Hospital, Turin, Italy; 3 Clinical Psychology Unit, Fondazione Policlinico Universitario A. Gemelli IRCCS, Rome, Italy; 4 Department of Cultures, Politics and Society, University of Turin, Turin, Italy; 5 Institute for Mater and Child Health, IRCCS Burlo Garofalo, Trieste, Italy; 6 UOSVD Psicologia Ospedaliera, P.O. Vito Fazzi, ASL Lecce, Lecce, Italy; 7 Pediatrics and Pediatric Hemato-Oncology Unit, SS Annunziata Hospital, Taranto, Italy; 8 LAD ETS Cure & Care in Pediatric Oncology, Policlinico Di Catania, Catania, Italy; 9 LAD ETS Cure & Care in Pediatric Oncology, Catania, Italy; 10 Faculty of Humanities, Department of Psychology and Health Sciences, Pegaso Telematic University, Naples, Italy; 11 Clinical Psychology Service, IRCCS AOU of Bologna, Bologna, Italy; 12 Department of Sciences of Public Health and Pediatrics, University of Turin, Turin, Italy; 13 Department of Biomedical, Metabolic and Neural Sciences, University of Modena and Reggio Emilia, Modena, Italy

**Keywords:** Professional quality of life, Burnout, Stress, Resilience, Pediatric onco-hematology, Rehabilitation professionals

## Abstract

**Background:**

Rehabilitation professionals in pediatric onco-hematology face intense emotional and relational demands that heighten vulnerability to burnout. Despite their key role in children’s recovery, this group remains underrepresented in psycho-oncology research.

**Objective:**

To assess burnout, compassion satisfaction, compassion fatigue, perceived stress, and resilience among rehabilitation professionals in Italian pediatric onco-hematology centers and to explore the organizational meanings underpinning their experiences.

**Methods:**

A mixed-methods multicenter study was conducted across eleven centers in the AIEOP network. Thirty professionals completed validated scales (ProQOL-5, Maslach Burnout Inventory, Perceived Stress Scale, Resilience Scale for Adults). Semi-structured interviews were analyzed using Automated Co-occurrence Analysis for Semantic Mapping (ACASM).

**Results:**

Participants reported high compassion satisfaction and moderate burnout, with 66.7% scoring high on depersonalization. Compassion fatigue correlated positively with emotional exhaustion (*ρ* = 0.45, *p* = 0.013) and perceived stress (*ρ* = 0.36, *p* = 0.048). Resilience did not correlate significantly with distress measures. ACASM identified two semantic dimensions—Relationship–Intervention and Users–Institution—indicating that emotional strain stems largely from institutional and relational dynamics rather than patient contact.

**Conclusions:**

Rehabilitation professionals experience a complex balance of meaning and fatigue. Organizational support, structured supervision, and participatory dialogue with management are crucial to sustain well-being and preserve care quality.

## Background

Burnout is a psychological condition arising from prolonged exposure to unmanaged work-related stressors, characterized by emotional exhaustion, depersonalization, and reduced personal accomplishment [1]. The World Health Organization classifies burnout as an occupational phenomenon rather than a medical condition, emphasizing its systemic and organizational origins [2]. Conceptually, burnout results from the interaction of occupational factors (e.g., workload, limited autonomy), personal traits (e.g., perfectionism, maladaptive coping), and organizational context (e.g., poor teamwork, inadequate recognition) [3–8].

Professionals exposed to continuous emotional engagement with individuals in need, such as healthcare and social service providers, are particularly susceptible to burnout [9, 10]. Pediatric onco-hematology is an especially demanding context, requiring both technical competence and sustained emotional involvement. Rehabilitation professionals, including physical therapists, support children and families throughout complex treatment trajectories, often becoming central emotional figures during recovery. While burnout has been extensively investigated among physicians and nurses, rehabilitation professionals remain underrepresented in the literature, despite experiencing comparable levels of exposure [11, 12]. These practitioners in fact engage in intense, emotionally charged interactions that combine physical care with relational and motivational support, yet their professional quality of life, coping strategies, and sources of resilience have not been systematically examined. Moreover, the few available studies on rehabilitation professionals have been conducted in general hospital or adult settings, providing little insight into the unique relational and organizational pressures of pediatric onco-hematology.

To address this gap, we conducted the multicenter Rehabilitation Burnout study (REBURN) within the Italian Association of Pediatric Hematology and Oncology (AIEOP) Rehabilitation Working Group [13], using a mixed-methods design to integrate quantitative psychometric data with insights from professionals’ narratives. Addressing this gap is critical to developing targeted preventive and institutional strategies for sustaining workforce well-being and care continuity in this highly specialized field.

## Methods

### Study design

A convergent mixed-methods design with an exploratory aim was employed. Quantitative and qualitative data were collected concurrently and analyzed independently, with integration occurring at the interpretive level. The quantitative component consisted of standardized self-report questionnaires assessing burnout, compassion satisfaction and fatigue, perceived stress, and resilience. The qualitative component consisted of semi-structured interviews analyzed using the Automated Co-occurrence Analysis for Semantic Mapping (ACASM) procedure.

The design was exploratory rather than explanatory, as no a priori causal or directional hypotheses were tested, and the qualitative findings were not used to explain or model specific quantitative associations. Instead, ACASM was used to identify the latent semantic dimensions organizing professionals’ narratives, which were then employed to contextualize and enrich the interpretation of the quantitative results. This approach allowed for an integrated understanding of psychological outcomes and organizational meaning-making without assuming causal precedence between methods [14, 15].

### Participants and recruitment

Participants were members of the Rehabilitation Working Group of the AIEOP [13] from several pediatric onco-hematology centers across Italy. Inclusion criteria were having signed a written informed consent form, being an Italian rehabilitation practitioner (physiotherapist, speech therapist, developmental neuro, and psychomotor therapist), having at least 1 year of work experience in pediatric onco-hematology. To clarify the roles of the rehabilitation professionals involved: in Italy, physiotherapists primarily address motor aspects, including neurological and orthopedic rehabilitation, often through play-based approaches. In contrast, the developmental neuro- and psychomotor therapist (TNPEE) focuses on supporting neurodevelopment, particularly the integration of neurological and psychomotor skills. The speech therapist is responsible for the assessment and rehabilitation of swallowing disorders (dysphagia), communication, speech and voice disorders caused by brain tumors, or chemotherapy and radiotherapy treatments. All enrolled participants completed the self-report questionnaires and took part in the interviews.

Recruitment took place from April to May 2024. In April, two psychologists of the research team attended a meeting of the AIEOP Rehabilitation Working Group and presented the aims of the study along with the informed consent form to invite participation in the study. The target sample of approximately 30 participants was determined a priori to ensure coverage across the eleven participating centers while remaining feasible for in-depth qualitative data collection. The aim was to enroll at least 85% of the rehabilitation professionals from the eleven participating centers. As there were thirty-five members of the AIEOP Rehabilitation Working Group from these centers, we estimated that thirty participants would provide acceptable representation, as this corresponds to 85.71% of the sample.

### Ethics

The study was approved by the University of Turin Bioethics Committee (decision Prot. No 0282438 dated 24.06.2024) in accordance with the principles of the Declaration of Helsinki. Voluntary written informed consent was obtained from all participants who agreed to participate in the study. All methods were carried out in accordance with relevant guidelines and regulations.

### Measures

Professionals who agreed to participate filled out a set of questionnaires including the Professional Quality of Life Scale (ProQOL-5), the Maslach Burnout Inventory (MBI), the Perceived Stress Scale (PSS-10), and the Resilience Scale for Adults (RSA).

The Italian version [16, 17] of the ProQOL-5 [18] was used to assess the professional quality of life of the study sample. It is a 30-item Likert scale ranging from “Never” to “Very often” (1–5 Likert scale). The ProQOL-5 (Professional Quality of Life Scale) has three subscales, Compassion Satisfaction (PROQOL_CSs), Burnout (ProQOL_CF_bo) and Secondary Traumatic Stress (ProQOL_CF_st); each with scores ranging from 10 to 50; according to the manual, scores ≤ 22 indicate low. Total scores of each scale were used to assess correlations between measures.

The MBI [19] measures burnout levels through twenty-two items rated on a seven point Likert scale, ranging from “Never” (0 points) to “Daily” (6 points). The Maslach Burnout Inventory has three subscales with relative cut-offs: Emotional Exhaustion (MBI_EE: low ≤ 18, moderate 19–26, high ≥ 27), Depersonalization (MBI_DEP: low ≤ 5, moderate 6–9, high ≥ 10), and Personal Accomplishment (MBI_PA: low ≤ 30, moderate 31–39, high ≥ 40). The Italian version of the Maslach Burnout Inventory, adapted and validated for the Italian population [20], was employed in the present study.

The PSS-10 [21] is a self-report measure designed to assess perceived stress levels through ten items on a Likert-type scale with response options ranging from “Never” (0 points) to “Very often” (4 points). The Perceived Stress Scale-10 (PSS-10) has a total score ranging from 0 to 40, with values of 0–13 indicating low stress, 14–26 indicating moderate stress, and 27–40 indicating high stress. Similar to what was done for the ProQOL-5, in the present study the PSS-10 score was used as an indicator for comparison with the other scales, rather than in relation to the standard cut-off points.

An Italian version of the PSS, previously applied in comparable populations, was employed [22].

The RSA [23] assesses intrapersonal and interpersonal protective factors that support individuals in adapting to life challenges. The scale consists of thirty-three items rated on a five-point Likert scale ranging from “Very often” (1 point) to “Never” (5 points). It includes four subscales measuring personal traits: Perception of Self (RSA_PS), Planned Future (RSA_PF), Social Competence (RSA_SC), and Structured Style (RSA_SS), and two subscales measuring interpersonal resources, i.e., Family Cohesion (RSA_FC) and Social Resources (RSA_SR). Consistent with the other questionnaires, the Italian version of the RSA was used [24]. The Resilience Scale for Adults (RSA) measures resilience and does not have strictly defined clinical cut-offs. In the present study, only the total score was considered for comparison with the other scales, without reference to any cut-off points.

### Interviews

In addition to the quantitative measures, thirty participants also took part in semi-structured online interviews, which were designed to capture more nuanced insights regarding their emotional experiences, coping strategies, and perceptions of burnout.

Five psycho-oncologists selected because they are part of the AIEOP Psychosocial Working Group conducted the interviews. The set of interview questions was developed and agreed upon in collaboration with experienced psychologists external to the working group and each participant was interviewed by a psychologist unknown to them and belonging to a different center than their own, to minimize potential bias. Only four burnout-related questions were identified for the analysis. The selection criterion was the presence of some form of standardization. Specifically, the selected questions consisted of items addressing different aspects and dimensions of burnout:

1. Reflective awareness: *from your point of view, what are the criticisms of your work?*
2. Organizational constraints and resources: *your institution in what does it facilitate and/or hinder you in organizing your service?*
3. Professionals’ agency: *do you have in mind proposals as to what your institution could do to help you?*
4. Shared professional identity: *what mission do you share with the institution which you work for?*

### Data analyses

Descriptive statistics were calculated to summarize the demographic characteristics of the sample. The median, mean, and standard deviation were calculated for the quantitative variables. Since the sample did not satisfy the assumption of normality, as determined by the Shapiro–Wilk test [25], non-parametric statistical methods were applied. The Shapiro–Wilk *W* statistics and corresponding *p*-values for all analyzed variables are reported in Tables 1 and 2. Spearman’s rank correlation was used to assess associations between variables, while the Mann–Whitney *U* test was conducted to evaluate differences between male and female participants, physiotherapists, and other rehabilitation professionals, and permanent and temporary staff. Additionally, the Kruskal–Wallis test was employed to compare differences across three geographical groups (North, Center, South). All statistical analyses were performed using R (version 4.1). Two-tailed *p*-values were reported, with statistical significance set at *p* < 0.05, and 95% confidence intervals (CI) were provided.

**Table 1:** Sample characteristics

Variables			Frequencies and %/mean and SD/median and IQR/range	*W*	*P*
Sex	Male		10 (33.3%)		
	Female		20 (66.7%)		
Age		Median	33 (IQR 7.7; range: 26–50)	0.902	0.010*
		Mean	34.3 (SD 6.3; range: 26–50)		
Region of residence	Northern Italy		23 (76.7%)		
	Central Italy		4 (13.3%)		
	Southern Italy		3 (10%)		
Marital status	Single/unmarried		13 (43.3%)		
	Divorced		1 (3.3%)		
	Married/civil partnership		16 (53.3%)		
Children	Yes		12 (40%)		
	No		18 (60%)		
Educational level	Postgraduate certificate		8 (26.7%)		
	Bachelor’s degree		13 (43.3%)		
	Master’s degree		8 (26.7%)		
	Degree + specialization		1 (3.3%)		
Profession	TNPEE		8 (26.7%)		
	Physiotherapists		19 (63.3%)		
	Speech therapists		3 (10%)		
Years of work		Median	9 (IQR 8.7; range: 3–27)	0.923	0.032*
		Mean	10.6 (SD 5.9; range: 3–27)		
Years of work in the oncology field		Median	4 (IQR 4; range: 1–21)	0.831	< 0.001***
		Mean	6.2 (SD 4.5; range: 1–21)		
Working hours per week		Median	36 (IQR 4.2; range: 12–56)	0.895	0.006**
		Mean	35.5 (SD 9; range: 12–56)		
Type of contract	Scholarship		4 (13.3%)		
	Freelance		9 (30%)		
	Fixed-term		1 (3.3%)		
	Permanent		16 (53.3%)		

*IQR* interquartile range → Intervallo interquartile, *SD* standard deviation → Deviazione standard W, Shapiro–Wilk test statistic for normality, *p*, *p*-value *p < 0.05, **p < 0.01, ***p < 0.001, indicate significant deviation from normalcy

**Table 2:** Results from questionnaires

Questionnaires	Mean, SD	Median, IQR	Range	*W*	*P*
PROQOL_CSs	38.4 (SD 4.8)	40 (IQR 7)	29–45	0.934	0.064
PROQOL_CF_bo	24.0 (SD 4.5)	23 (IQR 5.75)	17–35	0.944	0.120
PROQOL_CF_st	22.5 (SD 5.0)	22 (IQR 7)	13–32	0.984	0.910
MBI_EE	19.2 (SD 9.3)	18.5 (IQR 13.75)	4–36	0.963	0.374
13 (43.3%) low					
12 (40%) medium					
5 (16.7%) high					
MBI_DEP	13.5 (SD 4.8)	14 (IQR 6)	4–25	0.985	0.935
1 (3.3%) low					
9 (30%) medium					
20 (66.7%) high					
MBI_PA	32.5 (SD 5.9)	33.5 (IQR 5.75)	14–41	0.922	0.030*
3 (10%) low					
12 (40%) medium					
15 (50%) high					
MBI_TOT	65.3 (SD 15.5)	65.5 (IQR 19.75)	22–91		
PSS-10	20.1 (SD 5.4)	20 (IQR 5)	7–32	0.978	0.766
RSA_PS	17.8 (SD 2.2)	18 (IQR 2.75)	13–22		
RSA_PF	12.7 (SD 2.3)	12 (IQR 3)	9–19		
RSA_SC	17.6 (SD 2.34)	18 (IQR 2.75)	12–22		
RSA_SS	11.9 (SD 1.8)	12 (IQR 2)	6–15		
RSA_FC	18.5 (SD 2.3)	18.5 (IQR 2.75)	12–23		
RSA_SR	19.6 (SD 2.7)	19 (IQR 3.75)	15–25		
RSA_TOT	98.1 (SD 1.9)	85.5 (IQR 19.85)	73–113	0.920	0.027*

*PROQL_CSs* Compassion Satisfaction subscale of the Professional Quality of Life Scale, *ProQOL,* *PROQOL_CF_bo*, Burnout subscale of the ProQOL, *PROQOL_CF_st*, Secondary Traumatic Stress subscale of the ProQOL; *MBI_EE*, Emotional Exhaustion scale of the Maslach Burnout Inventory (MBI), *MBI_DEP* Depersonalization scale of the MBI, *MBI_PA* Personal Accomplishment scale of the MBI, *MBI_TOT* total score of the Maslach Burnout Inventory reflecting overall burnout, *PSS* total score on the Perceived Stress Scale (PSS), reflecting overall perceived stress, *RSA_PS* Perception of Self-subscale of the Resilience Scale for Adults (RSA), *RSA_PF* Perception of the Future subscale of RSA, *RSA_SC* Social Competence subscale of RSA, *RSA_SS* Structured Style subscale of RSA, *RSA_FC* Family Cohesion subscale of RSA, *RSA_SR* Social Resources subscale of RSA, *RSA_TOT* Total score on the Resilience Scale for Adults (RSA), representing individual resilience levels. *W* Shapiro–Wilk test statistic for normality, *p* *p*-value. *p < 0.05, indicate significant deviation from normalcy

The Automated Co-occurrence Analysis for Semantic Mapping (ACASM) procedure [14, 15] was used to identify the semantic structure underlying how professionals describe and conceptualize burnout. This procedure involved two stages [26], using T-Lab software [27].

In the first stage, the corpus was segmented into Elementary Context Units (ECUs)—small text portions that balance thematic interpretation. Segmentation followed a paragraph-based criterion: the algorithm split the text every 250 words, with each ECU ending at a punctuation mark (.,!, or?). If an ECU exceeded 2000 characters, it was cut at the last word within the length limit. To reduce lexical variation, all relevant lemmas were mapped to their etymological root (e.g., “cared” and “cares” were lemmatized as “to care”). Words lacking semantic content—such as stop-words, auxiliary verbs, and empty or indicative words—were excluded. Additionally, the 5% most frequent lexical forms were removed to reduce noise and highlight meaningful semiotic patterns. This process yielded a refined subset of the most prevalent lemmas, balancing computational feasibility with the ability to detect meaningful patterns in the data. The ECUs were then condensed into a digital matrix, where each cell indicated the presence (1) or absence (0) of a specific lemma (columns) within a given ECU (rows).

In the second stage, a correspondence analysis (COR) was applied to this matrix to uncover the semantic structure underlying the narratives [28]. Unlike the original ACASM procedure, Cluster Analysis was not performed, because here the focus of the research team was on mapping the semantic structures organizing the narratives rather than grouping them into themes. The COR reorganized the relationships between lemmas within a multidimensional factorial space, revealing oppositional patterns among categories. The two resulting factors each consisted of two polarities: the positive polarity was characterized by patterns of co-occurring lemmas, while the negative polarity was defined by their mutual absence. Higher or lower test values indicated stronger saturation of a given polarity, whereas values closer to zero reflected weaker saturation. The resulting factorial space thus served as an operational representation of the text’s semantic structure, with each factorial dimension capturing a distinct semantic component.

Finally, the factors were interpreted through a consensus procedure within the research team [26]. Each member independently assigned interpretations based on the characteristic lemmas of each factor; these interpretations were then discussed until agreement on a final label was reached.

## Results

### Sample characteristics

A total of thirty rehabilitation professionals working in pediatric onco-hematology across Italy participated in the study. The sample included speech therapists, TNPEE, and physiotherapists, working in eleven pediatric onco-hematology centers across Italy. The median age of respondents was 33 years (IQR [26–50]), and 66.7% (*n* = 20) were women. 76.7% (*n* = 23) of the sample lived in Northern Italy, 53.3% (*n* = 16) were married or in a civil relationship, and 60% (*n* = 18) had no children. Sixty-three percent (*n* = 19) of participants were physiotherapists, 27% (*n* = 8) and the 10% (*n* = 3) were TNPEE and speech therapists, respectively. Forty-three percent (n = 13) of the therapists held a Bachelor’s degree. Regarding work experience, the median number of years worked was 9 [IQR (3–27)], while the median number of years worked in pediatric onco-hematology was 4 [IQR (1–21)]. Fifty-three percent (*n* = 16) of the sample had a permanent work contract, and the median number of working hours per week was 36 [IQR (12–56)].

Complete demographic characteristics of the participants are reported in Table 1.

### Measures

Quantitative analyses describe the psychological profile of rehabilitation professionals (Table 2). Participants reported high levels of compassion satisfaction (mean = 38.4 ± 4.8). Moderate levels of burnout and perceived stress were observed across the sample. Depersonalization scores were high in two-thirds of respondents (66.7%). Emotional exhaustion scores showed variability, while personal accomplishment remained moderately high (mean = 32.5 ± 5.9).

Correlational analyses are reported in Table 3. Compassion fatigue—particularly its burnout component—was positively correlated with emotional exhaustion (*ρ* = 0.45, *p* = 0.013) and perceived stress (*ρ* = 0.36, *p* = 0.048). Compassion satisfaction was negatively correlated with burnout (*ρ* = − 0.58, *p* < 0.001) and positively correlated with personal accomplishment (*ρ* = 0.48, *p* = 0.008). Resilience scores were generally elevated, particularly in structured coping styles, family cohesion, and self-perception, but did not show significant correlations with burnout or stress measures.

**Table 3:** Correlation matrix between demographic information and scale scores

Correlation matrix											
		1	2	3	4	5	6	7	8	9	10
1. AGE	Rho di Spearman	–									
	*p*	–									
2. AGE JOB	Rho di Spearman	0.914***	–								
	*P*	< 0.001	–								
3. PROQOL_CSs	Rho di Spearman	0.024	0.100	–							
	*p*	0.900	0.598	–							
4. PROQOL_CF_bo	Rho di Spearman	0.052	0.001	−0.575***	–						
	*p*	0.783	0.995	< 0.001	–						
5. PROQOL_CF_st	Rho di Spearman	−0.00	0.005	−0.370*	0.368*	–					
	*p*	0.990	0.980	0.044	0.046	–					
6. MBI_EE	Rho di Spearman	−0.00	0.053	−0.138	0.449*	0.600***	–				
	*p*	0.990	0.782	0.466	0.013	< 0.001	–				
7. MBI_DEP	Rho di Spearman	0.088	0.059	−0.225	0.317	0.420*	0.460*	–			
	*p*	0.644	0.756	0.232	0.088	0.021	0.011	–			
8. MBI_PA	Rho di Spearman	0.277	0.243	0.476**	−0.329	0.050	0.288	−0.029	–		
	*p*	0.138	0.195	0.008	0.076	0.793	0.123	0.877	–		
9. PSS_TOT	Rho di Spearman	−0.00	−0.04	−0.164	0.364*	0.512**	0.241	0.251	−0.210	–	
	*p*	0.962	0.803	0.387	0.048	0.004	0.199	0.180	0.265	–	
10. RSA_TOT	Rho di Spearman	0.013	0.065	−0.242	0.160	0.076	0.003	0.074	−0.229	0.134	–
	*p*	0.946	0.734	0.198	0.398	0.690	0.988	0.696	0.223	0.481	–

*p < 0.05, **p < 0.01, ***p < 0.001

*AGE participant age*, AGE JOB, years of work experience in the current professional role; *PROQL_CSs* Compassion Satisfaction subscale of the Professional Quality of Life Scale (ProQOL); *PROQOL_CF_bo* Burnout subscale of the ProQOL; *PROQOL_CF_st* Secondary Traumatic Stress subscale of the ProQOL; *MBI_EE* Emotional Exhaustion scale of the Maslach Burnout Inventory (MBI); *MBI_DEP* Depersonalization scale of the MBI; *MBI_PA* Personal Accomplishment scale of the MBI; *PSS* Total score on the Perceived Stress Scale (PSS), reflecting overall perceived stress; *RSA_TOT* Total score on the Resilience Scale for Adults (RSA), representing individual resilience level

**Fig. 1:**
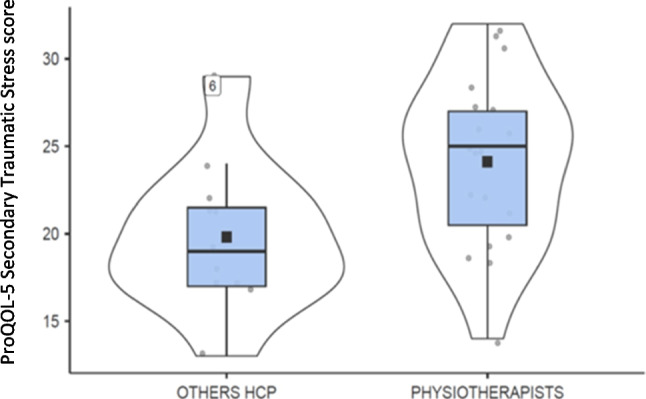
Distribution of ProQOL-5 Secondary Traumatic Stress (PROQOL_CF_st) scores between Physiotherapists and other Health Care Professionals (HCP). *Y*-axis represents ProQOL-5 Secondary Traumatic Stress (PROQOL_CF_st) scores. Abbreviations: HCP = Health Care Professionals

Non-parametric group comparisons provided additional information on burnout-related variables. The Mann–Whitney test showed no statistically significant sex differences across the scales, although depersonalization scores were higher in male participants (mean = 16.1 ± 5.4) than in females (mean = 12.3 ± 4.0). When professional roles were compared, physiotherapists reported significantly higher secondary traumatic stress (ProQOL-CF_st) than other rehabilitation professionals (*U* = 49.5; *p* < 0.05; mean = 24.1 ± 4.9 vs. 19.8 ± 4.3; see Fig. 1). No significant differences were observed with respect to employment type (permanent vs. temporary). Regional comparisons across the North, Center, and South of Italy showed higher compassion satisfaction among professionals working in Northern Italy (*p* < 0.005), together with higher levels of burnout, secondary traumatic stress, and emotional exhaustion (*p* < 0.05 to *p* < 0.01). Perceived stress scores showed a similar pattern (*p* < 0.005). Given the modest sample size, these results should be interpreted with caution.

### Interviews

The analysis of the interviews through the COR produced two factorial dimensions (Table 4) which are interpreted by the research team as follows:

- *Factor 1. Relationship–intervention*. The negative pole of this factor groups lemmas referred to the healthcare service of therapists offered in terms of relationships, thus highlighting the emotional, professional, and relational component played by the actors (e.g., role, recognition, project). Positive pole lemmas, on the other hand, refer to the concept of the healthcare service of therapists offered in terms of medical intervention (e.g., service, hospitalization, inpatient care).
- *Factor 2. Users–hospital management*. The negative pole of this factor includes lemmas that refer to relationships between rehabilitation professionals and the target audience (e.g., quality, rehabilitation, life). On the other hand, the positive pole of this factor groups lemmas referred to the relationships within the hospital; specifically, between health workers and the hospital management group (e.g., organize, manage, room)*.*

**Table 4:** Characteristic lemmas of factorial dimensions

Factor 1				Factor 2			
Relationship		Intervention		Users		Hospital management	
Pole (−)	*V* test	Pole (+)	*V* test	Pole (−)	*V* test	Pole (+)	*V* test
Role	−13.0414	Intervention	4.3592	Service	−8.2104	Certainly	2.7174
Recognition	−10.2177	Quality	3.8052	Quality	−7.6430	Week	2.6392
To recognize	−6.9194	Inpatient care	3.3627	Life	−6.0425	Difficulty	2.5918
Project	−4.4051	Life	3.3490	Project	−5.5482	Face	2.2711
Inside	−4.3799	Service	3.2653	Inpatient care	−4.8875	Day	2.0304
Therapist	−3.8093	To improve	2.7554	Improve	−4.4897	Laborious	2.2058
Demand	−3.6386	Hospital management	2.7514	Role	−4.1150	Report	2.2018
To facilitate	−3.5880	Stuff	2.6260	High	−3.8137	Organizing	2.1760
Association	−3.0102	To lose	2.5703	Company	−3.4554	Nice	2.1723
Difficult	−2.9337	To search	2.4578	Imagine	−3.1659	Situation	2.1360

**Fig. 2:**
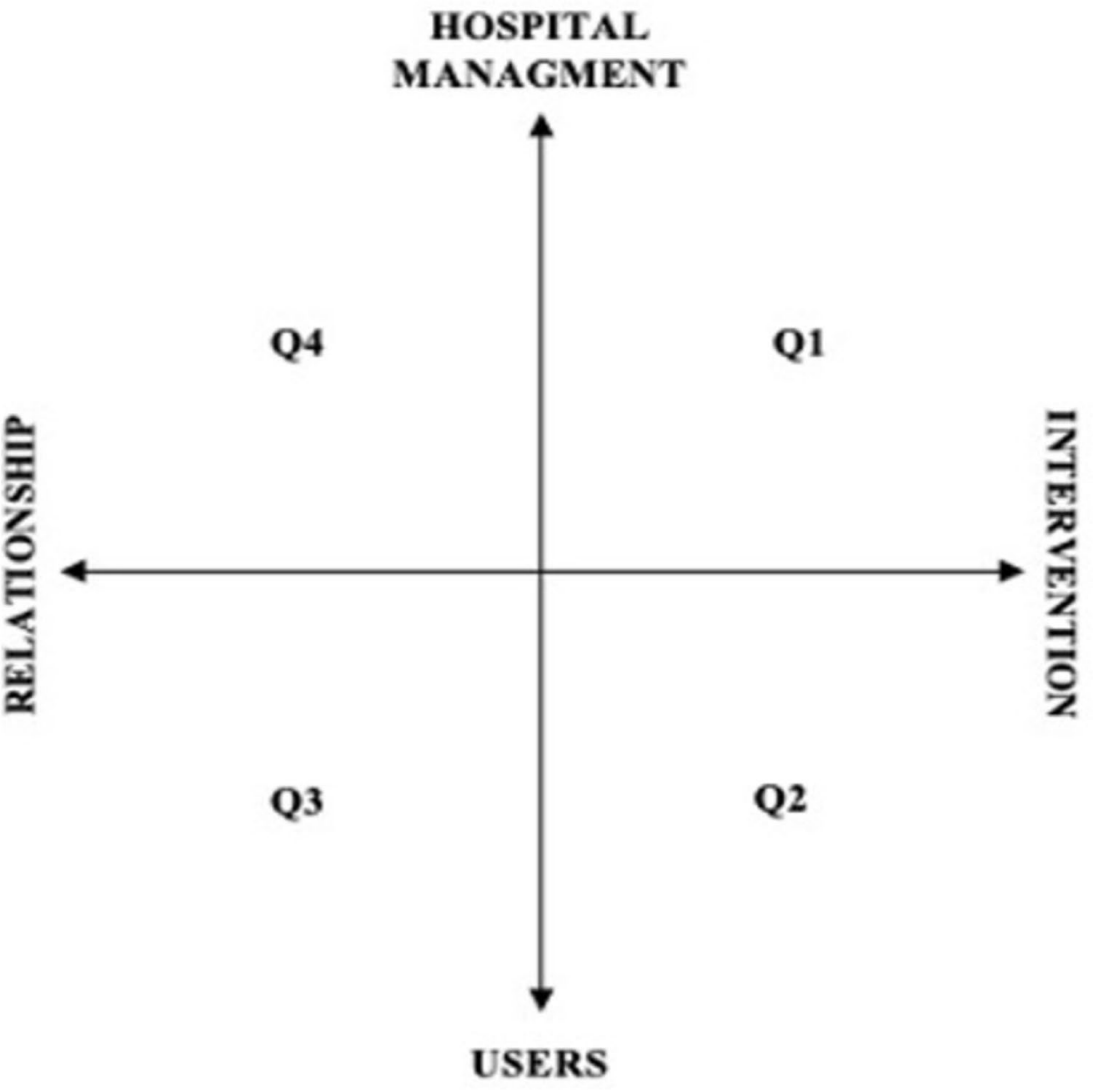
Semantic space defined by Factor 1 (*x*-axis) and Factor 2 (*y*-axis)

The intersection of these two factors defined four quadrants (Fig. 2), which represented the semantic structure behind the answers received at each item: Quadrant 1 (Q1), Quadrant 2 (Q2), Quadrant 3 (Q3), and Quadrant 4 (Q4).

- *Quadrant 1 (Q1)*: defined the rehabilitation intervention as a health provision shaped by internal dynamics between colleagues and hospital management.
- *Quadrant 2 (Q2)*: outlined the rehabilitation intervention as a health provision as well, but focusing on the relationship with the users.
- *Quadrant 3 (Q3)*: interpreted the rehabilitation intervention as a relationship with users.
- *Quadrant 4 (Q4)*: reads the rehabilitation intervention as a relationship, which unfolded predominantly within professionals; it focused on the internal relations between colleagues and the company.

**Fig. 3:**
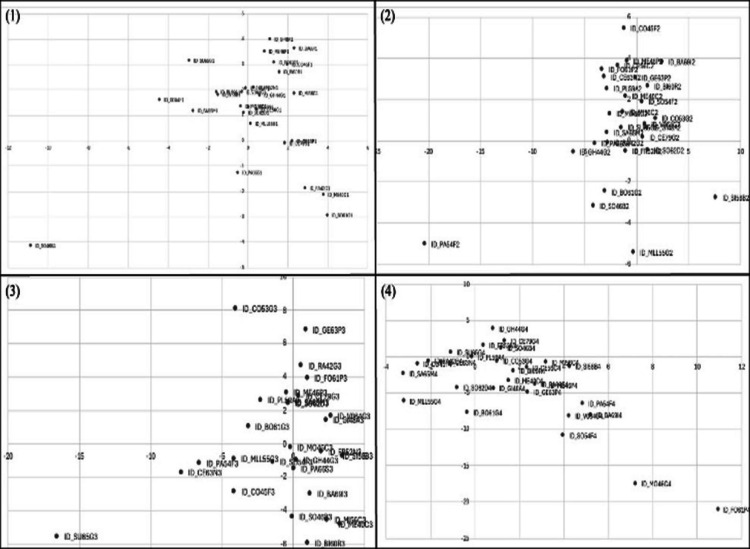
Respondents’ positioning for each item

By analyzing the placement of respondents at each question and therefore item (Fig. 3), it was possible to formulate the following considerations:

- *Item 1*: The saturation in this case prevailed in two quadrants; Q1 counting 12 respondents and Q4 counting 9.
- *Item 2*: The most saturated quadrant was Q4 (12 respondents). It emerged also that none of the answers polarized the first factor (Relationship–Intervention), all hovering around the value 0.
- *Item 3*: In this instance, the responses are more disparate. As could be seen in the Cartesian plane, all the respondents are approximately evenly distributed in the four quadrants. Q2 and Q3 being the most saturated quadrant, with 8 respondents each. Instead, Q1 and Q4 were occupied by 6 respondents each.
- *Item 4*: In this case, most respondents were distributed in Q2 (16); the remaining respondents were mostly in Q3 (8) and Q4 (4), leaving Q1 with just one.

## Discussion

This study provides the first comprehensive overview, within the Italian context, of the psychological profile of rehabilitation professionals working in pediatric onco-hematology. The findings reveal a multidimensional emotional experience, highlighting the coexistence of compassion satisfaction, stress, and burnout in a highly specialized and emotionally charged care setting. This complexity underscores that well-being in pediatric onco-hematology cannot be reduced to the absence of burnout but reflects a dynamic equilibrium between emotional engagement and psychological protection.

Participants reported moderately high levels of Compassion Satisfaction, suggesting that many perceive their work as meaningful and rewarding despite the inherent challenges of pediatric onco-hematology [29]. Nevertheless, the simultaneous presence of burnout symptoms and secondary traumatic stress demonstrates the profound emotional cost of sustained empathic involvement with children and families facing life-threatening illness [30]. The negative association between compassion satisfaction and burnout suggests that maintaining professional meaning may act as a protective factor against emotional exhaustion, while the positive correlation with Personal Accomplishment indicates that perceived efficacy and purpose mitigate fatigue and moral distress [31].

A particularly salient result is the high prevalence of depersonalization, with 66.7% of respondents classified as high risk. Even among professionals with relatively low emotional exhaustion, depersonalization emerged as a dominant coping strategy, functioning as an adaptive form of emotional self-preservation in the face of chronic exposure to suffering [32]. This dual configuration—simultaneous engagement and withdrawal—captures the paradox of caregiving in pediatric onco-hematology: clinicians may sustain compassion and commitment while emotionally detaching to prevent psychological overload [1]. Prior research has shown that such patterns, while protective in the short term, may over time erode empathy and relational quality, ultimately impacting both provider well-being and patient experience [23].

Perceived stress levels were moderate but clinically relevant and significantly correlated with both emotional exhaustion and compassion fatigue, supporting stress as a central pathway through which emotional and organizational pressures manifest [33]. The lack of significant association between stress and overall resilience suggests that resilient individuals are not immune to contextual strain, reinforcing the need for system-level approaches rather than relying solely on individual coping capacity.

Consistent with evidence from other health professions [34, 35], resilience appeared to function as a background protective trait rather than being directly associated with emotional distress. However, subdomain analyses revealed high scores in Structured Style, Family Cohesion, and Perception of Self, highlighting valuable personal and interpersonal resources. These findings point to the potential benefit of targeted interventions—such as peer supervision, reflective groups, and family-informed resilience training—that leverage these existing strengths.

No significant gender differences emerged, yet the trend toward higher depersonalization among men may warrant attention for its implications on relational style and caregiving behaviors. Physiotherapists showed higher secondary traumatic stress than other rehabilitation specialists, plausibly reflecting their closer physical contact and prolonged patient interaction [36]. Regional variability—with less favorable scores among professionals in Northern Italy—suggests potential differences in institutional support, workload distribution, or access to psychosocial resources, though these patterns must be interpreted cautiously given the small and not balanced sample.

The ACASM procedure complemented the quantitative findings by identifying the latent organizing dimensions of professionals’ discourse on burnout. Two dominant semantic structures emerged, each defined by a dichotomous opposition: *Relationship vs. Intervention* and *Users vs. Hospital Management*. Since semantic structures ground and shape the content of representations, different positions within the semiotic space reflect different ways of making sense of burnout. The respondents’ positioning thus instantiated these underlying structures, reflecting the image that individuals and groups—in this case, therapists—hold of themselves and their relationship to the surrounding context. The analysis of each item revealed distinct sense-making dynamics.

The first item showed that most respondents framed workplace difficulties in terms of relationships within the organization—both as a place of intervention (Q1, *n* = 12) and as a place of relationship (Q4, *n* = 9). This suggests that perceived difficulties do not arise in relation to users, but rather in the possibility of relating to hospital staff and management. Notably, users were largely absent from professionals’ accounts of workplace challenges: when describing difficulties, respondents thought of themselves in relation to the organization as a superordinate structure, rather than in relation to those they serve. This represented a paradox, as respondents questioned the organization without recognizing themselves as part of it. The second item, concerning how the institution hinders the organization of care, was similarly saturated in Q4. Respondents again identified relationships—both within the organization and with users—as the primary obstacle. Importantly, responses did not polarize along the first factor (Relationship vs. Intervention), suggesting that respondents did not perceive the organization as either a place of performance or a place of genuine relationship. Instead, the organization appeared as an empty entity, stripped of its relational dimension—a perception that may, in turn, make engagement with it more difficult. The third item produced more dispersed positioning. When asked what the organization could do to facilitate their work, respondents drew on varied frames of reference—relationships with users, relationships with the institution, performance-oriented care, or relational care—with no clear convergence. This lack of a shared direction suggests that practitioners were uncertain about the core source of their difficulties, rendering intervention difficult: without a clear sense of what needs to change, the organization is left without a meaningful path forward. The fourth item revealed that most respondents recognized their professional mandate in the provision of healthcare (Q2). However, thirteen respondents were distributed across the remaining quadrants, and eight specifically framed their mission in relational terms rather than in terms of health outcomes. This suggests that a meaningful subgroup of professionals understands their role primarily as a relational one, rather than as a service aimed at promoting well-being—a noteworthy distinction with implications for how care is conceived and delivered.

Overall, the REBURN study depicts a workforce that is deeply motivated yet emotionally burdened, finding meaning in care but constrained by organizational stressors. Addressing burnout in this context requires multilevel interventions—from structured psycho-oncological supervision and reflective practice to organizational strategies that foster recognition, communication, and shared purpose [37, 38]. Promoting a culture where caregiver well-being is viewed as integral to care quality is essential to ensuring sustainability and excellence in pediatric onco-hematology rehabilitation.

## Clinical and research implications

These findings emphasize that burnout prevention in pediatric onco-hematology must extend beyond frontline clinical staff to include rehabilitation professionals—those who sustain children’s functional recovery and family adaptation. Emotional strain in this group arises less from patient contact and more from organizational and relational contexts, where recognition, role clarity, and communication are pivotal.

*At the clinical level*, psycho-oncologists should be systematically integrated into rehabilitation teams to provide structured supervision, reflective groups, and resilience workshops. Regular debriefings and institutional acknowledgment of emotional labor can help prevent the progressive detachment signalled by high depersonalization scores.

*At the organizational level*, healthcare managers, together with occupational health specialists, should embed participatory feedback mechanisms, ensuring that rehabilitation professionals are active partners in shaping care processes. Developing clear professional pathways and institutional cultures that value teamwork, flexibility, and psychological safety may enhance motivation, retention, and burnout prevention.

*At the research level*, future work should employ longitudinal designs to track burnout trajectories and intervention efficacy, exploring how resilience interacts with institutional belonging and professional identity over time. Cross-national collaborations could clarify whether the observed patterns reflect broader systemic dynamics in pediatric onco-hematology rehabilitation.

By promoting a culture that values both patient and staff well-being, institutions can safeguard the sustainability and quality of pediatric onco-hematology rehabilitation care.

## Study limitations

The main limitations of this study are the small sample size, which may reduce generalizability and statistical power, and the lack of formal quantification of inter-rater agreement in the ACASM analysis, which may affect robustness. The inclusion criterion of at least one year of work experience in pediatric onco-hematology represents a further limitation. Although this threshold was adopted to ensure exposure beyond initial training and role-transition phases, it may have limited the inclusion of professionals with longer cumulative career trajectories, in whom burnout and stress processes may be more pronounced. Future studies using stratification by years of experience and longitudinal designs are warranted to better capture the accumulation and evolution of burnout over time. The heterogeneity of professional roles may have obscured role-specific patterns, and the cross-sectional design precludes causal inference. Longitudinal studies are needed to examine the temporal dynamics of burnout and the protective role of resilience. Future research should further explore the role of marital status, previously considered by other research [12], and extend the investigation to other professional groups, such as psycho-oncologists, for whom evidence remains limited.

## Conclusion

This study illuminates the psychological experience of rehabilitation professionals in pediatric onco-hematology, revealing a complex emotional landscape marked by high compassion satisfaction, moderate stress, and salient burnout symptoms—particularly depersonalization. These findings highlight the paradox of caregiving in emotionally demanding settings: professionals derive deep meaning from their work yet often rely on emotional distancing to preserve psychological stability [39].

The absence of significant associations between resilience and distress-related variables suggests that resilience alone may not buffer against the cumulative effects of chronic exposure to suffering. This underscores the importance of addressing organizational and relational factors that shape professionals’ well-being.

Targeted preventive strategies—such as structured individual and group psycho-oncological support, regular supervision, and the promotion of supportive team cultures—can mitigate burnout and enhance resilience [40, 41]. Embedding these measures within multidisciplinary and participatory frameworks can strengthen both staff cohesion and patient care. Institutions should actively foster environments where professionals feel valued, recognized, and involved in decision-making, transforming workplaces from sources of strain into spaces of shared purpose.

Future research, ideally using longitudinal and cross-national designs, should further delineate the interplay between institutional climate, personal resources, and emotional health. Ultimately, cultivating a “culture of care for the caregivers”—in which staff well-being is considered integral to care quality—is essential to sustain both workforce vitality and excellence in pediatric onco-hematology rehabilitation.

## Data Availability

Data available on request from the corresponding authors.

## Abbreviations

ACASM: Automated Co-occurrence Analysis for Semantic Mapping
AIEOP: Italian Association of Pediatric Hematology and Oncology (Associazione Italiana di Ematologia e Oncologia Pediatrica)
CI: Confidence interval
COR: Correspondence analysis
ECU: Elementary Context Unit
IQR: Interquartile range
MBI: Maslach Burnout Inventory
MBI_DEP: Depersonalization subscale of the Maslach Burnout Inventory
MBI_EE: Emotional Exhaustion subscale of the Maslach Burnout Inventory
MBI_PA: Personal Accomplishment subscale of the Maslach Burnout Inventory
MBI_TOT: Total score of the Maslach Burnout Inventory
PSS: Perceived Stress Scale
PSS-10: Perceived Stress Scale–10 item version
ProQOL: Professional Quality of Life Scale
ProQOL_CSs: Compassion Satisfaction subscale of the Professional Quality of Life Scale
ProQOL_CF_bo: Burnout subscale of the Professional Quality of Life Scale
ProQOL_CF_st: Secondary Traumatic Stress subscale of the Professional Quality of Life Scale
REBURN: Rehabilitation Burnout Study
RSA: Resilience Scale for Adults
RSA_FC: Family Cohesion subscale of the Resilience Scale for Adults
RSA_PF: Planned Future subscale of the Resilience Scale for Adults
RSA_PS: Perception of Self subscale of the Resilience Scale for Adults
RSA_SC: Social Competence subscale of the Resilience Scale for Adults
RSA_SR: Social Resources subscale of the Resilience Scale for Adults
RSA_SS: Structured Style subscale of the Resilience Scale for Adults
RSA_TOT: Total score of the Resilience Scale for Adults
TNPEE: Developmental neuro and psychomotor therapist
*W*: Shapiro–Wilk test statistic
